# Comparing the Cell Dynamics of Tree-Ring Formation Observed in Microcores and as Predicted by the Vaganov–Shashkin Model

**DOI:** 10.3389/fpls.2020.01268

**Published:** 2020-08-14

**Authors:** Valentina Buttò, Vladimir Shishov, Ivan Tychkov, Margarita Popkova, Minhui He, Sergio Rossi, Annie Deslauriers, Hubert Morin

**Affiliations:** ^1^ Département des Sciences fondamentales, Université du Québec à Chicoutimi, Chicoutimi, QC, Canada; ^2^ Laboratory for Integral Studies of Forest Dynamics of Eurasia, Siberian Federal University, Krasnoyarsk, Russia; ^3^ Environmental and Research Center, South China Botanical Garden, Chinese Academy of Sciences, Guangzhou, China; ^4^ College of Forestry, Northwest Agriculture and Forestry University, Yangling, China; ^5^ Key Laboratory of Vegetation Restoration and Management of Degraded Ecosystems, Guangdong Provincial Key Laboratory of Applied Botany, South China Botanical Garden, Chinese Academy of Sciences, Guangzhou, China

**Keywords:** modeling, xylogenesis, cell diameter, timings, cambial cells, black spruce, boreal forest, growth rate

## Abstract

New insights into the intra-annual dynamics of tree-ring formation can improve our understanding of tree-growth response to environmental conditions at high-resolution time scales. Obtaining this information requires, however, a weekly monitoring of wood formation, sampling that is extremely time-intensive and scarcely feasible over vast areas. Estimating the timing of cambial and xylem differentiation by modeling thus represents an interesting alternative for obtaining this important information by other means. Temporal dynamics of cambial divisions can be extracted from the daily tree-ring growth rate computed by the Vaganov–Shashkin (VS) simulation model, assuming that cell production is tightly linked to tree-ring growth. Nonetheless, these predictions have yet to be compared with direct observations of wood development, i.e., *via* microcoring, over a long time span. We tested the performance of the VS model by comparing the observed and predicted timing of wood formation in black spruce [*Picea mariana* (Mill.)]. We obtained microcores over 15 years at 5 sites along a latitudinal gradient in Quebec (Canada). The measured variables included cell size and the timing of cell production and differentiation. We calibrated the VS model using daily temperature and precipitation recorded by weather stations located on each site. The predicted and observed timing of cambial and enlarging cells were highly correlated (*R* ^2^ = 0.8); nonetheless, we detected a systematic overestimation in the predicted timing of cambial cells, with predictions delayed by 1–20 days compared with observations. The growth rate of cell diameter was correlated with the predicted growth rate assigned to each cambial cell, confirming that cell diameter developmental dynamics have the potential to be inferred by the tree-ring growth curve of the VS model. Model performances decrease substantially in estimating the end of wood formation. The systematic errors suggest that the actual relationships implemented in the model are unable to explain the phenological events in autumn. The mismatch between the observed and predicted timing of wood formation in black spruce within our study area can be reduced by better adapting the VS model to wet sites, a context for which this model has been rarely used.

## Introduction

Modeling permits the description of complex biogeochemical processes that occur in nature (Danis et al., 2012), particularly as a suite of factors drive tree-growth response to climate. Tools such as MAIDENiso, TreeRing2000, and the Vaganov–Shashkin (VS) model are mechanistic models for predicting tree growth that account for the endogenous and exogenous factors shaping tree growth and productivity (Vaganov et al., 2006; Danis et al., 2012). Among these models, the VS model requires the smallest number of inputs. Furthermore, these inputs include data that are widely used and easily available, such as tree-ring width chronologies and daily mean precipitation and temperature. The availability of these data has led to an increased use of the VS model, which can now be parameterized using a user-friendly interface, the VS-oscilloscope (Shishov et al., 2016) or new MATLAB version of the model (Anchukaitis et al., 2020). Nonetheless, the intra-annual predictions of the VS model continue to lack validation with long-term field observations that, unlike tree-ring chronologies, are scarce.

The main prediction of the VS model is the daily tree-ring growth rate, which is obtained by integrating three partial growth rates based on day-length (photoperiod), temperature, and soil water content. The variation of these environmental factors over the entire year, including the growing season, affects xylem cell production and development to result finally in different wood increments (Vaganov et al., 2006; Balducci et al., 2016). The VS model has been applied to simulate long-term climate responses based on the variation of tree-ring width chronologies at a regional scale; study locations include the southeastern United States (Anchukaitis et al., 2006; Evans et al., 2006) and the Tibetan Plateau (He et al., 2017; Yang et al., 2017). Parameterization of the model is necessary to account for the endogenous components of tree-growth response and represents a critical but necessary step of the modeling process, a step that can provide important information about tree growth at the local scale (Evans et al., 2018; Tychkov et al., 2019).

The core of tree-ring growth is cambial activity, and the VS model has been developed to establish not only the start and end of the growing season but also the timing of xylem cell production and the final size of these cells (Popkova et al., 2018). This particular aspect of the VS model has heightened interest in its application with the abundance of literature discussing intra-annual tree-ring dynamics within the cambial zone and xylem; these dynamics are related to both endogenous (developmental patterns and hormones) and exogenous (weather and seasonality) factors (Buttò et al., 2020). The variation of xylem cell traits, i.e., cell diameter and cell wall thickness, provides important information about the trade-off between hydraulic safety and efficiency, a factor that allows plants to adapt and survive in a changing environment (Hacke et al., 2017). Cell traits depend on the temporal dynamics (duration, rate) of their differentiation phases, and environmental factors, i.e., temperature and precipitation, all of which have an influence that varies over the growing season (Fonti et al., 2010; Cuny et al., 2019). By disentangling the effects of environmental factors on tree-ring development at a daily scale, the VS model may have the potential to simulate xylem cell differentiation, even though the existing version of the model focuses mainly on cambial cell production.

The dynamics of cell development—computed *via* the tree-ring growth rate curves of the VS model—are emergent properties that must be validated *via* observations of xylem formation. The interpretation of these emergent properties of the models should benefit from the observations (Cook and Pederson, 2011); however, unlike the traditional inter-annual tree-ring widths, long intra-annual chronologies of secondary growth are rare and data sets that are limited to a couple of years of observation do not ensure a complete picture of tree-growth response to environmental factors as these responses are often nonlinear (Rossi, 2015). Apart from the study of Popkova et al. (2018), which was based on three years of observation of wood formation, to our knowledge no study has compared the timing of cell development simulated by a VS model with data obtained directly through repeated observations of xylogenesis using microcores.

To validate the VS model predictions with observations, we used our existing 15-year, weekly scaled chronologies of wood formation from across the boreal forest of Quebec, Canada. We aimed to compare the predicted and observed timing of wood growth at both a tree-ring and xylem-cell resolution by using microcores collected from black spruce (*Picea mariana* Mill.). Black spruce is the dominant species in the Canadian boreal forest and it grows within a great diversity of stand structures, from Alaska to Newfoundland. In Quebec, the distribution of this species extends to 58°N and forms extensive, closed forests in northeastern North America, including some of the wettest and coldest boreal forest stands. The ubiquity of black spruce leads to a very diversified tree-growth response to climate, reflecting the role of various local environmental drivers (Walker and Johnstone, 2014; Nicault et al., 2015).

For model calibration at the tree-ring scale, we relied on the existing literature and field information to assess the performance of the VS model for predicting the variability of factors and parameters that affect black spruce growth. Then, we used observations of xylogenesis to validate the timing of wood formation at the tracheid scale, i.e., cell scale, for which variability is well represented by our long time series of observations.

## Methods

### Study Sites and Tree Selection

Samples were collected from five sites in the coniferous boreal forest of Quebec (Canada) along a latitudinal gradient stretching between 48°N and 53°N (**Table 1**). The sites Simoncouche (SIM) and Bernatchez (BER) are located in the balsam fir [*Abies balsamea* (L.) Mill.)]–white birch (*Betula papyrifera* Marsh.) bioclimatic domain, while Mistassibi (MIS) and Camp Daniel (DAN) lie in the black spruce–moss bioclimatic domain. Mirage (MIR), the northernmost site, lies in the black spruce–lichen domain and is characterized by a lower tree density and growth than the more southern sites. Mean annual temperature ranges between −3.4 and 1.9°C, with the southernmost and northernmost sites being the warmest and the coldest, respectively (**Table 1**). Precipitation ranges from 626 to 906 mm along the latitudinal gradient with drier conditions toward the north (**Table 1**). At each site, we selected ten dominant or co-dominant trees, avoiding individuals having polycormic stems, evident parasite damage, reaction wood, or partially dead crowns (**Table 1**).

**Table 1:** Location, climatic conditions, and tree characteristics at the five study sites, ordered in terms of latitude.

**Site**	**Latitude (N)**	**Longitude (W)**	**Altitude(m asl)**	**Annual temperature (** **°** **C)**	**Annual precipitation (mm)**	**DBH (cm)**	**Height (m)**
**SIM**	48°13′	71°15′	338	1.9	906	20.4 ± 2.4	16.1 ± 1.2
**BER**	48°51′	70°20′	611	0.1	886	21.1 ± 3.7	17.3 ± 1.8
**MIS**	49°43′	71°56′	342	0.3	755	19.6 ± 2.8	18.3 ± 1.1
**DAN**	50°41′	72°11′	487	−1.3	735	18.5 ± 2.9	16.6 ± 2.2
**MIR**	53°47′	72°52′	384	−3.4	626	19.6 ± 3.0	13.1 ± 1.2

DBH, diameter at breast height. Annual statistics for temperature and precipitation were calculated from 1950–2016 data using the ANUSPLIN algorithm (McKenney et al., 2011).

### Climate Measurements

Precipitation, temperature, and soil water content at 30 cm soil depth were collected by automatic weather stations equipped with CR10X data loggers (Campbell Scientific Corporation, Canada); these stations were installed in a forest gap within each site. We averaged hourly measurements to obtain daily time series. We filled any minor data gaps caused by short-term technical problems using the ANUSPLIN model (Hutchinson et al., 2009; Hopkinson et al., 2011; McKenney et al., 2011).

### Xylem Formation Dynamics

Microcores were collected weekly or fortnightly between April and October (2002–2016) from 10 individuals per site. Sampling was performed using a surgical bone needle (2002–2007) or Trephor (2007–2016). Sampling at MIR took place from 2012 to 2016. Microcores were dehydrated through successive series of immersions in ethanol and D-limonene. The microcores were embedded in paraffin, cut into 8 µm transversal sections, and stained with cresyl violet acetate (0.16% in water). Xylem cell development was detected by counting the number of cells undergoing each stage of cell differentiation. We counted cambial and xylem cells along three radial lines and identified the differentiation stages of (I) enlargement, (II) cell wall thickening and lignification and (III) mature tracheids. During wood formation, cambial derivatives start dividing and differentiating in xylem and phloem cells by increasing in size. The maturation of the xylem cells is achieved once secondary wall deposition and lignification are completed (Plomion et al., 2001). Cells undergoing different differentiation stages are identified by their different shape, size, color and glistening under polarized light. Cambial cells are irregularly shaped and smaller than xylem cells (Skene, 1969). Due to the different reactions of the components of cell walls to cresyl violet acetate, enlarging cells show pinkish coloration while cells undergoing secondary wall deposition are stained in violet, turning blue when mature (Deslauriers et al., 2003). Mature cells glisten under polarized light.

We estimated the daily sequence of dividing, differentiating, and maturating cells by fitting generalized additive models (GAM) with splines to the number of cells counted for each sampling day, thereby assessing cell production and the timing of division and differentiation of each tree-ring cell at a daily scale (Cuny et al., 2013). Timing represented the day of the year (DOY) when each cell entered into a new developmental stage (Buttò et al., 2019). Accordingly, we estimated the DOY in which each cambial cell stopped dividing and differentiated into an enlarging xylem cell i.e. timing of enlargement, and the DOY in which each cell stopped enlarging and started cell wall deposition i.e. timing of secondary wall deposition and lignification. The duration of enlargement was computed as the difference between the timing of secondary wall deposition and the timing of enlargement for each cell. The period considered as wood formation spanned from the DOY when the number of cambial cells increased in spring to the DOY when the last formed tracheids in the tree-ring was fully mature.

### Wood Anatomy

In summer 2017, we collected additional microcores from 10 individuals per site (Rossi et al., 2006). We prepared these microcores using the abovementioned procedure, stained them in safranin (1% in water), and permanently fixed the samples on slides using Permount™. We obtained pictures of the transversal sections using a camera fixed on an optical microscope at a magnification of 20×. Radial lumen diameter and cell wall thickness (single wall) were measured for all the study years (15 for all sites except for MIR, for which we had 5 years of observation) using WinCELL (Regent Instruments, Canada). We calculated the tracheidograms of cell diameter, relying on their relative position across the tree ring, and fit our results with GAMs to obtain values representative of each site and year, accounting for cell production as assessed by xylogenesis monitoring (Buttò et al., 2019). We assessed the growth rates of cells as a ratio between cell diameter and the duration of enlargement; the value was scaled between 0 and 1 to be compared with the nondimensional tree-ring growth rate computed by the VS model (**Table 2**).

**Table 2:** Glossary of all terms linked to tree growth and cell temporal dynamics.

**ID**	**Description**	**Units**
**Timing of cell division**	Day of the year in which a cell starts dividing	DOY (Julian days)
**Timing of cell enlargement**	Day of the year in which a cell starts enlarging	DOY (Julian days)
**Cell growth rate**	Growth rate of cell diameter	µm per day
**Partial growth rate (photoperiod, water, temperature growth rates)**	Growth rate linked to environmental factors; their integration produces the tree-ring growth rate	Nondimensional (min 0, max 1)
**Tree-ring growth rate**	The main output of the VS model and represents the daily tree-ring growth rate	Nondimensional (min 0, max 1)
**Cambial cell growth rate**	Growth rate corresponding to the production of a new cambial cell	Nondimensional (min 0,max 1)

### Tree-Ring Time-Series Analysis

We measured tree-ring width using WinCELL (Regent Instruments Inc., Canada) on histological samples obtained from ten microcores collected at all five sample sites. To compare trees with different growth rates, we detrended the chronologies, removing the effects of tree age, genetic growth potential, microsite characteristics, and stand history (Cook et al., 1990). We applied 67% cubic splines with a 1/2 cut-off time-series length to detrend and produce standardized chronologies for the 2002–2016 period using the *detrend* and *chron* functions of the dplR package in R (Bunn, 2008).

### VS Model Calibration and Validation

We used standardized tree-ring width series to calibrate the VS model for 2002–2016 using the VS-oscilloscope (Shishov et al., 2016). We performed parameterization to avoid any contradictions with field observations and available information for the sites (Tychkov et al., 2019). Accordingly, the calibration of the environmental parameters in the VS oscilloscope, was performed considering the measures of our weather stations during the different moments of the growing season, that we identified by means of the xylogenesisis monitoring. Site-specific parameters were calibrated considering site features, while for parameters linked to black spruce ecology, like root deepness, we relied on literature references to check if the values we established were realistic. Simulations of daily soil water content were improved by activating the soil melting block in the VS-oscilloscope, a parameter that was designed originally to include permafrost melting (Shishov et al., 2016). As we were dealing with wet sites, we used this block to consider the marked amount of water released by snowmelt at the start of the growing season. Nonetheless, the current version of VS model does not estimate soil thawing and soil moisture for the dormancy, because these values are assumed to be constant.

For each site, we evaluated the effect of the environmental factors on tree-ring growth *via* graphical interpretation of the partial growth rates patterns provided by the VS model simulations (Vaganov et al., 2006). The graphical representation of the daily average partial growth rates simulated by the VS model allows to determinate the most limiting factor to growth at daily scale, which corresponds to factors linked to the lowest growth rate (Shishov et al., 2016). Indeed, tree-ring growth rate is a function of three partial growth rates that depend on daily temperature, soil water content, and photoperiod; these factors are integrated into the tree-ring growth rate and represent an important result of the model simulations (**Table 2**). The start of the growing season as predicted by the model depends on the crossing of a critical threshold for all three partial growth rates; this occurs when each environmental factor activates or permits tree-ring growth (Vaganov et al., 2006). In addition to the minimum temperature for growth to start (T_min_), the sum of temperature for growth initiation (T_beg_) can be parameterized, in order to consider the forcing needed for cambial resumption. In the current version of VS-oscilloscope, the end of growth occurs when the integral growth rate of the tree ring falls below a critical threshold (critical growth rate Vcr) (Tychkov et al., 2019). We obtained the general pattern of the partial growth rates using the default arguments of the function geom_spline from the R’s package “ggformula” (Kaplan and Pruim, 2020).

We computed the cambial cell growth rate, i.e., the average growth rate corresponding to cambial cells produced each year, and then determined their timing of division and enlargement following Popkova et al. (2018) (**Table 2**). The protocol proposed by Popkova et al. (2018) extrapolates the timing of cell division and enlargement by the tree-ring growth rate, assuming that the production of a new cambial cell occurs only once the previous cells have left the cambial zone. Cell production would indeed occur without overlapping. The observed number of cells produced each year is necessary to calculate temporal dynamics of cell production, which we obtained through our observations of the microcores.

We assessed the robustness of the VS model simulations using the actual and predicted tree-ring width indices; we relied on the Pearson correlation (R) and root mean square error (RMSE). We retained only models showing a significant R (p < 0.05), producing the smallest RMSE to minimize the variance of simulated indices and to select the simulation with the smallest average prediction error (James et al., 2013). The model validation involved comparing the simulations (named “predicted data”) with our results from the microcores (named “observed data”). Pearson correlations and linear regressions served to compare the predicted and observed data. We transformed the data when necessary to meet the assumption of normality, and we fit LOESS functions (span = 0.7) to compare the general patterns visually.

## Results

### Climate Along the Latitudinal Gradient

For the 2002–2016, our weather stations recorded warmer monthly mean annual temperatures than the long-term average (**Tables 1** and **3**). For this same period, mean annual precipitation was higher in the north (715 mm, MIR) and lower in the south (622 mm, SIM), with a greater northward inter-annual and daily intra-annual variability during wood formation (**Table 3**). However, mean annual and mean daily soil water content decreased from south to north, spanning from 0.17 V/Vs at MIR to 0.36 V/Vs at SIM over the year (**Table 3**). Daily soil water content during wood formation ranged from 0.17 to 0.42 V/Vs at DAN and BER, respectively.

**Table 3:** Average weather conditions and soil water content (with standard deviation) for 2002–2016 as recorded by the weather stations established at the study sites; data for MIR covers 2012–2016.

	**During the year**			**During wood formation**		
**Site**	Mean temperature (°C)	Mean precipitation(mm)	SWC(V/Vs)	Mean daily temperature (°C)	Mean daily precipitation (mm)	Daily SWC (V/Vs)
SIM	2.1 ± 0.8	622 ± 106	0.33 ± 0.04	14.5 ± 4.4	2.45 ± 5.2	0.33 ± 0.05
BER	0.4 ± 0.9	704 ± 105	0.36 ± 0.07	13.5 ± 4.0	2.93 ± 5.8	0.42 ± 0.08
MIS	1.0 ± 0.9	794 ± 145	0.25 ± 0.06	14.6 ± 3.9	3.33 ± 6.5	0.26 ± 0.07
DAN	−1.0 ± 1.0	708 ± 221	0.18 ± 0.03	13.4 ± 3.8	3.36 ± 6.5	0.17 ± 0.04
MIR	-2.6 ± 0.7	715 ± 126	0.17 ± 0.02	13.1 ± 4.0	3.79 ± 6.0	0.21 ± 0.05

Wood formation occurred from May to September, although specific dates varied between sites and years (see **Table 4** for details). SWC, soil water content.

### Duration and Rate of Xylem Growth

For all sites, correlations between the observed and predicted tree-ring width indices were positive and highly significant (SIM and MIS: *P* < 0.05, *N* = 15; BER, DAN: *P* < 0.01, *N* = 15; MIR: *P* < 0.01, *N* = 5) and *R*-values ranged from 0.54 to 0.90 (**Supplementary Figure S1**). RMSE ranged between 0.06 (DAN) and 0.1 (MIR), staying below the RMSE threshold of 0.3 and attesting to the good fit between the indices and the simulated results (**Supplementary Figure S1**). Fixed parameters of temperature for tree growth ranged from 4 to 29°C, showing a 1–5°C difference depending on the site (**Supplementary Table S1**). Minimum and maximum soil moisture levels were similar along the entire latitudinal gradient, although MIR had the lowest maximum soil moisture (**Supplementary Table S1**).

**Figure 1:**
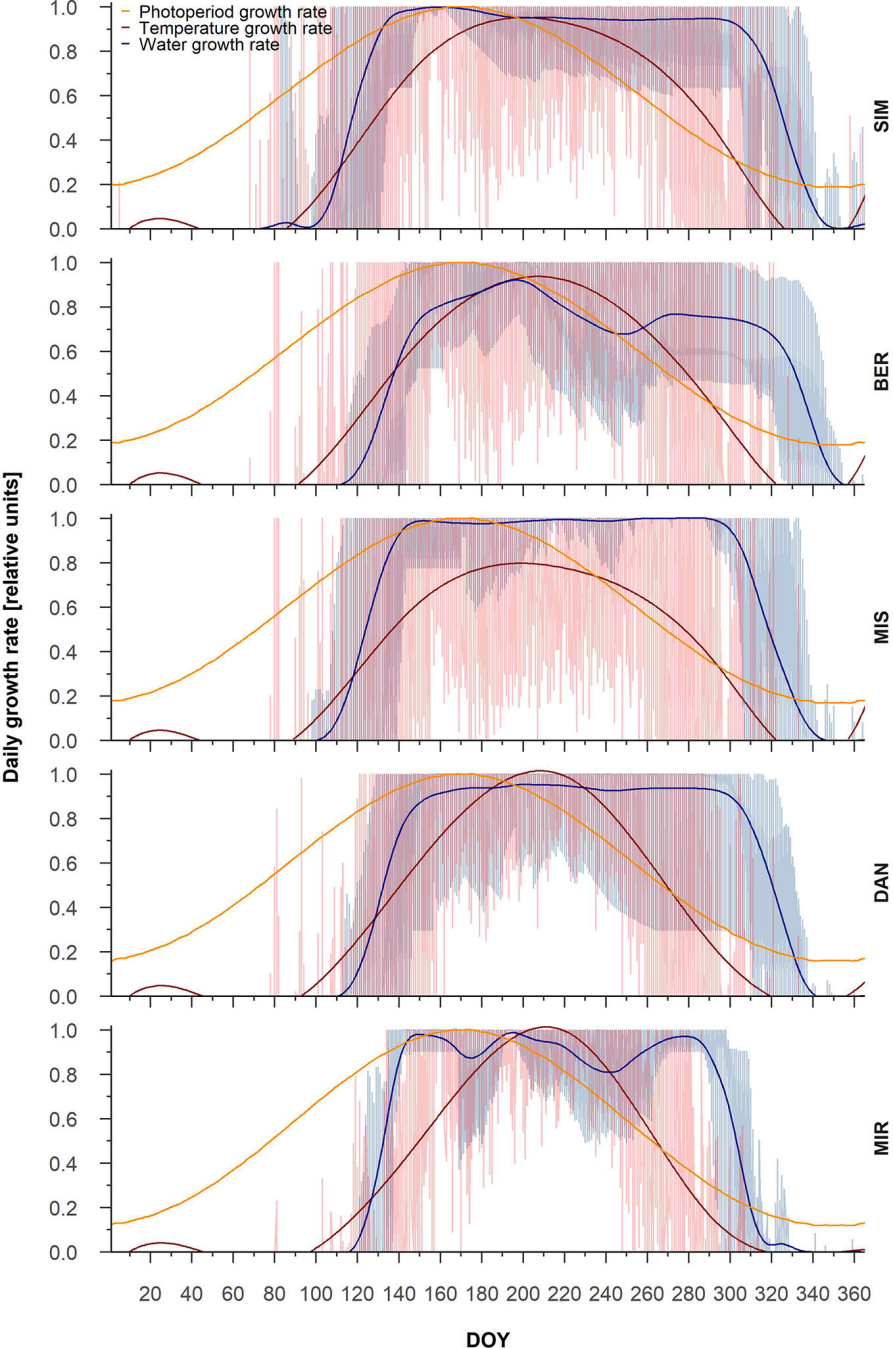
Intra-annual partial tree-ring growth rates linked to photoperiod (yellow), temperature (red), and water (blue). General trends for the three partial growth rates are represented by splines. The lowest partial growth rate represents the most limiting factor for each DOY.

Partial growth rates showed marked inter-annual variability, in particular for temperature growth rate (**Figure 1**). In general, the temperature growth rate peaked at the end of August (DOY 240), while the photoperiod growth rate peaked around the summer solstice (DOY 170) (**Figure 1**). The maximum temperature growth rate was highest at SIM, where it reached 0.8 relative units, and decreased to 0.62 at the northernmost site, MIR. The water growth rate reached 1, the maximum value, at SIM, MIS, and DAN, around the middle of July (DOY 200) for SIM and MIS and 20 days later at DAN. At MIR, the water growth rate peaked around DOY 240, although it never attained the maximal value (**Figure 1**).

**Figure 2:**
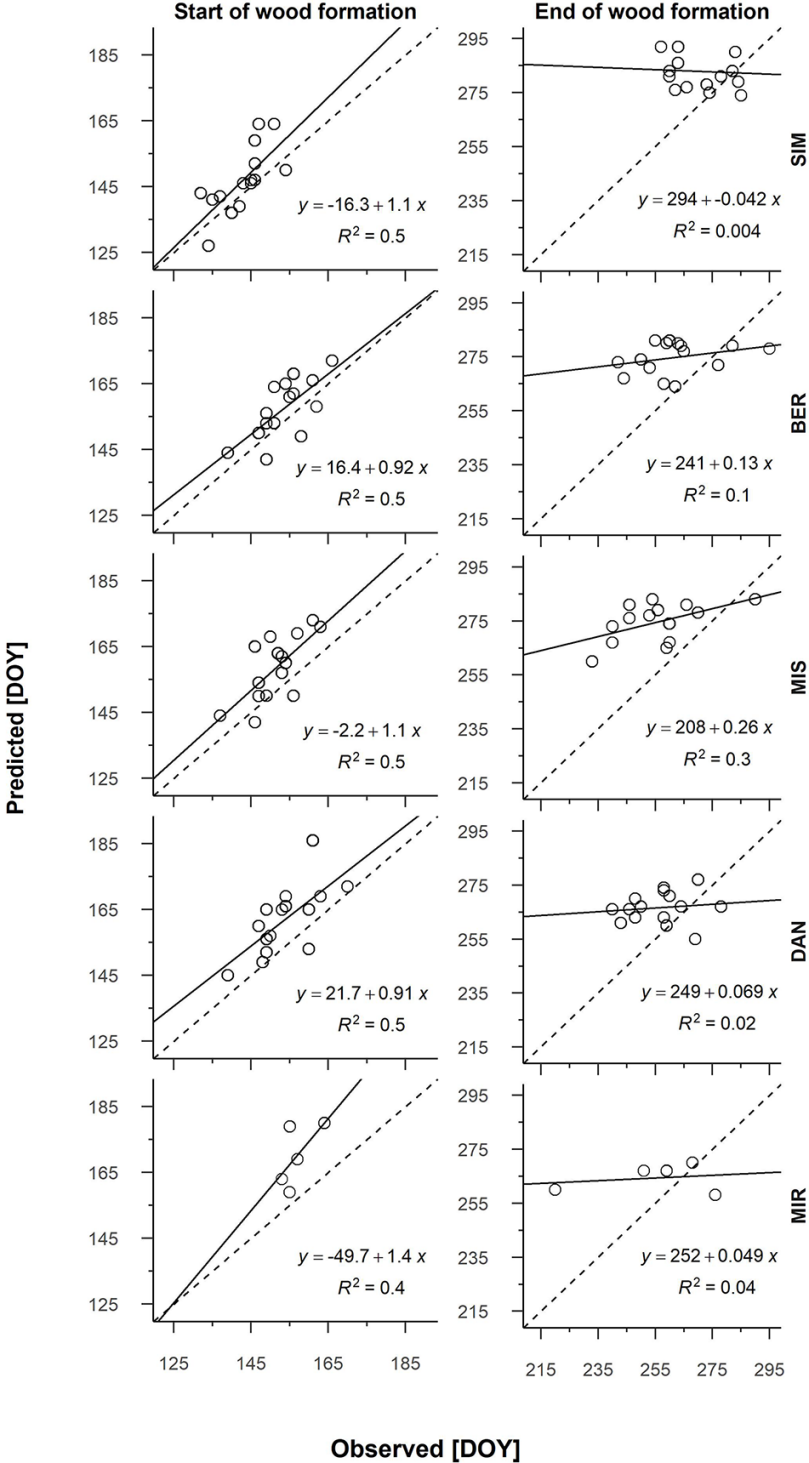
Linear regression of the predicted and observed start and end of wood formation for all sites and years (2002–2016; 2012–2016 for MIR). Each graph has a dashed 1:1 line. Sites are organized along the latitudinal gradient with the southernmost site (SIM) as the top row.

From the partial growth rate patterns (**Figure 1**), wood formation started when temperatures were suitable for the resumption of cambial activity, which according to predictions, occurred between the end of May and the onset of June. Cambial activation was earliest at the southernmost site (SIM, DOY 143) and latest in northernmost site (MIR, DOY 157) (**Figure 2**, **Table 4**). The predicted start of wood formation occurred 4–13 days after the observed start (**Figure 2**, **Table 4**). The linear relationships between the predicted and observed start of the wood formation produced *R* ^2^ values that ranged from 0.4 (MIR) to 0.5 (DAN) and showed a systematic overestimation for the start of wood formation (**Figure 2**). The end of wood formation—occurring when the photoperiod was the most limiting factor—was predicted latest for the southernmost site (SIM, on average DOY 267) and earliest for the northernmost site (MIR, on average DOY 255, **Table 4**). The predicted end of wood formation was generally overestimated with wood formation lasting 9–19 days longer than observed (**Table 4**). The observed end of wood formation showed a higher inter-annual variation of up to 2 weeks, whereas the predicted end of the growing season varied on average 6 days over the years (**Table 4**). The simulated and observed ends of wood formation showed a weak relationship, having *R* ^2^ values of only 0.02 (DAN) to 0.3 (MIS) (**Figure 2**).

**Table 4:** Start and end of wood formation (DOY) including inter-annual variation as determined by observations of xylogenesis and that simulated by the VS model.

	**Start**		**End**	
**Site**	Observed	Predicted	Observed	Predicted
**SIM**	143 ± 6.1	147 ± 9.7	267 ± 12.2	283 ± 6.7
**BER**	154 ± 6.6	157 ± 8.6	262 ± 13.6	275 ± 5.7
**MIS**	151 ± 6.4	158 ± 9.6	256 ± 13.6	275 ± 6.8
**DAN**	153 ± 7.6	162 ± 10.1	257 ± 10.7	267 ± 5.8
**MIR**	157 ± 3.9	170 ± 8.6	255 ± 19.7	264 ± 4.8

### Model Parameters and Environmental Properties

**Figure 3:**
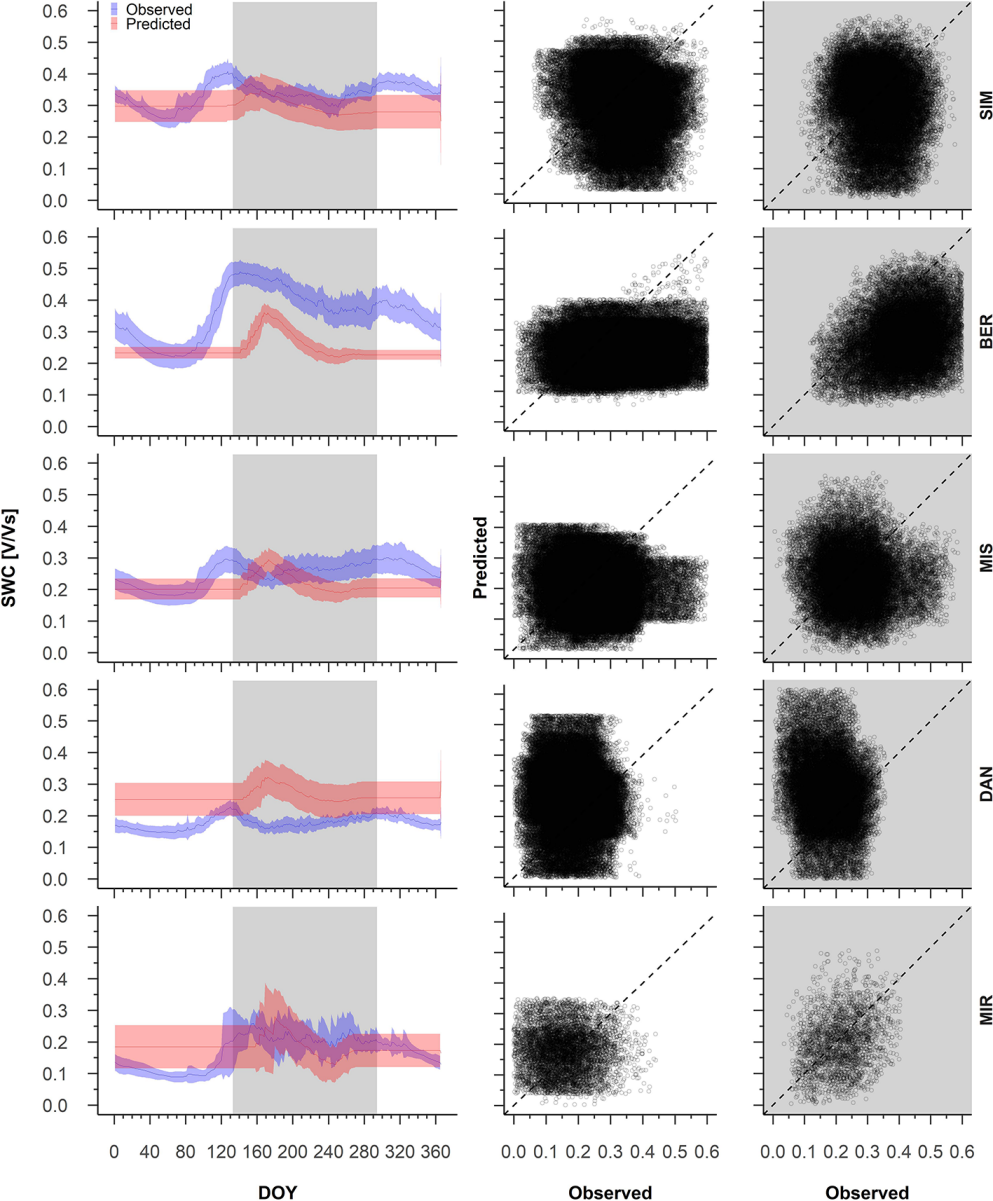
From the left to the right, average pattern of predicted (red) and observed (blue) soil water content with 95% confidence intervals (left), observed and predicted variations of soil water content during dormancy (middle) and during growing period (right). The white background on the graphs represents the dormancy period, whereas the shaded background represents the period of wood formation; Predicted versus observed soil water content for the dormant and wood formation periods. Sites are organized according to latitude (the southernmost site (SIM) as the top row of graphs, the northernmost (MIR) is on the bottom row).

Pearson correlations between the predicted and observed daily soil water content ranged between 1 and −1 (**Supplementary Table S2**). During dormancy, an average 16% of the simulated years produced a strong correlation (*R* > 0.4) between the predicted and observed daily soil water content (**Supplementary Table S2**). MIS showed the best performances, where 33% of the years showed a strong and positive correlation between the predictions and observations. We observed the lowest correlations at SIM, where the predicted and observed soil water content correlated strongly in only 7% of the years (**Supplementary Table S2**). The model simulations for winter resulted in a constant soil water content that was either an underestimate or overestimate depending on the year and site (**Figure 3**).

The observed soil water content showed a large intra-annual variation, in particular at the more southern sites (**Figure 3**). At SIM and BER, soil water content varied in winter between 0.2 and 0.4 V/Vs, culminating at the end of April (DOY 121, **Figure 3**). From the beginning of May, increased variability in soil water content mirrored a decrease in the observed soil water content, which dropped from 0.5 V/Vs at SIM and 0.6 V/Vs at BER to 0.3 V/Vs at both sites during the first week of October (**Figure 3**). During the second half of October, soil water content for these sites began to increase slightly and produced a smaller peak in mid-November. The soil water content at MIS and DAN varied little, peaking on DOY 121, although at a much lower intensity than at other sites; MIS and DAN soil water content varied between 0.2 and 0.3 V/Vs and 0.1 V and 0.2 V/Vs, respectively (**Figure 3**). In contrast, the predicted soil water content at all sites remained constant until DOY 162, generally underestimated at the southern sites (SIM, BER, and MIS), and overestimated at DAN (**Figure 3**).

During wood formation, we noted a strong correlation between the observed and predicted soil water content, with 32% of years having a correlation *R* > 0.4, whereas MIS only showed 7% of the years above this strong correlation threshold (**Supplementary Table S2**). We observed the best performances at MIR and BER where correlations between the predicted and observed summer soil water content were 60, and 80%, respectively (**Supplementary Table S2**). In most years, the predicted soil water content was overestimated at DAN and underestimated at SIM and BER (**Figure 3**). At MIS and MIR, the predicted vs. observed data points fell on the 1:1 intercept, although we also observed a marked variability (**Figure 3**).

### Variation in the Timing of Wood Formation Along the Latitudinal Gradient

**Figure 4:**
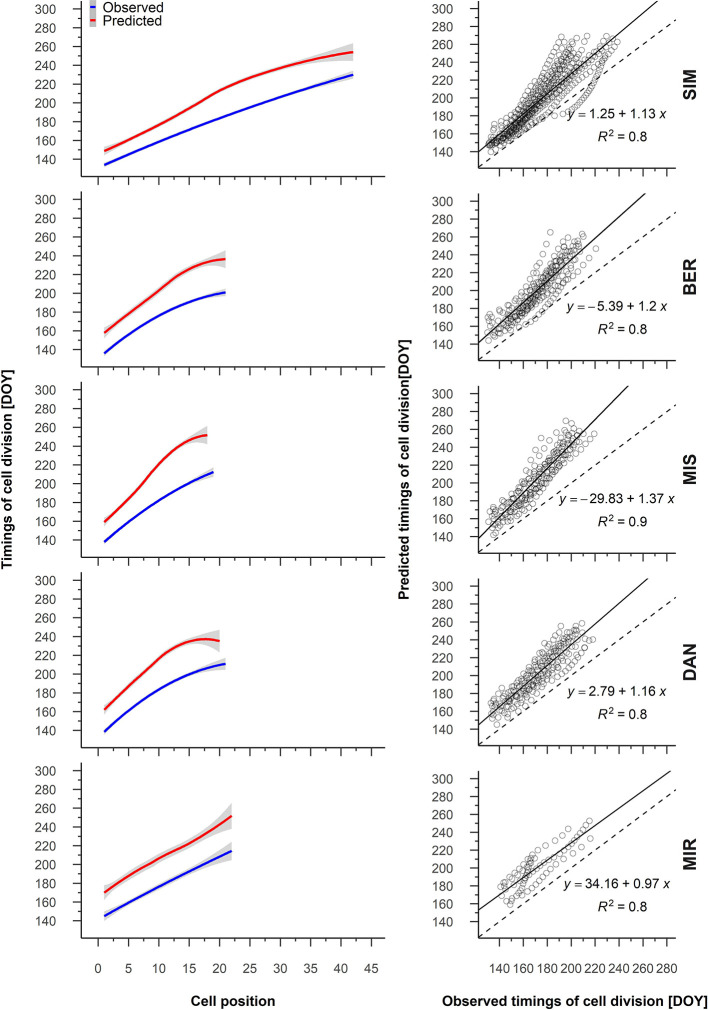
From the left to the right, average patterns for observed (blue) and predicted (red) timing of cell division in relation to cell position, gray shading represents the 95% confidence interval (left); regression between the predicted and observed timing of cell division (right). Sites are organized according to latitude (the southernmost site (SIM) as the top row of graphs, the northernmost (MIR) is on the bottom row).

For all sampled years, the predicted and observed timing of cell division and cell enlargement were highly correlated (*R* > 0.9, **Supplementary Table S2**). On average, the general pattern of the predicted timing of cell division showed a constant trend for cell positions 19 to 2 for DAN and MIR, respectively (**Figure 4**). Nevertheless, the predicted and observed timing for cambial cells demonstrated a strong linear relationship and *R* ^2^ values between 0.8 and 0.9 (**Figure 4**).

**Figure 5:**
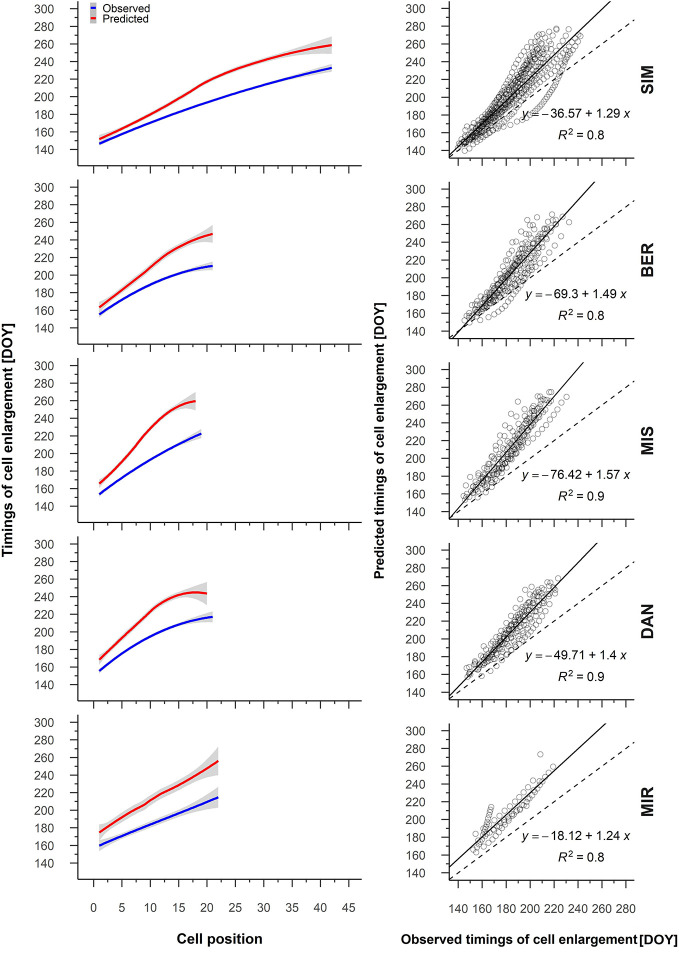
From the left to the right, average patterns for observed (blue) and predicted (red) timing of cell enlargement in relation to cell position, gray shading represents the 95% confidence interval (left); regression between the predicted and observed timing of cell enlargement (right). Sites are organized according to latitude (the southernmost site (SIM) as the top row of graphs, the northernmost (MIR) is on the bottom row).

We obtained similar strong correlations and relationships for the predicted and observed timings of cell enlargement (**Supplementary Table S2**, **Figure 5**); at the beginning of the growing season, however, the differences between the observed and predicted timings of cell enlargement were initially quite small—from 1 to 2 days depending on the site—and increased consistently with cell position, attaining a difference of 40 DOY (**Figure 5**).

**Figure 6:**
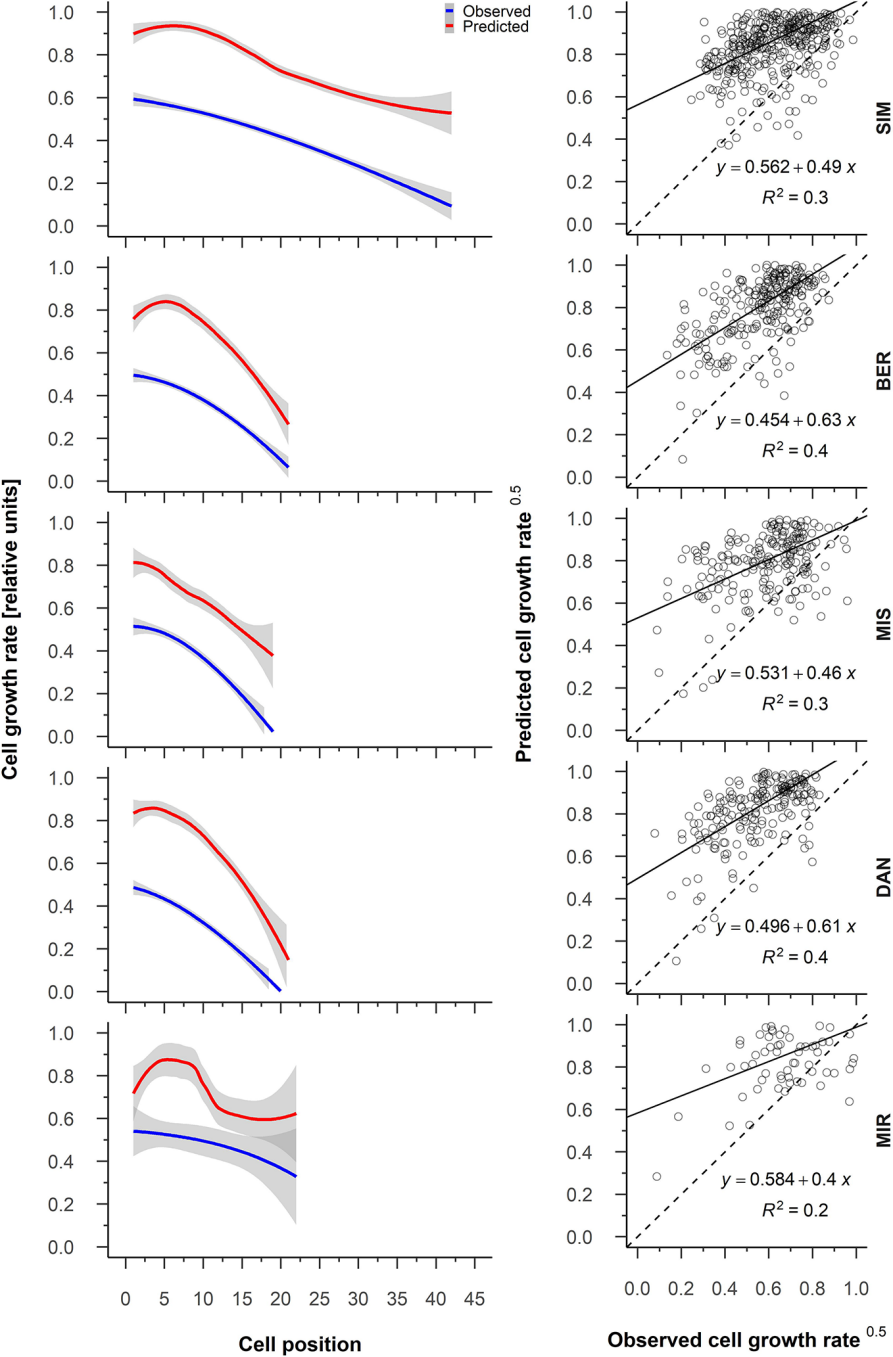
From the left to the right, average patterns for observed (blue) and predicted (red) cell growth rates in relation to cell position, gray shading represents the 95% confidence interval (left); regression between the predicted and observed cell growth rate (right). Sites are organized according to latitude (the southernmost site (SIM) as the top row of graphs, the northernmost (MIR) is on the bottom row).

The relationship between predicted cambial cell growth rate and observed cell growth rate for all five sites showed *R* ^2^ values of 0.4 at MIR, DAN, and SIM to 0.3 at MIS (**Figure 6**). The predicted cambial cell and observed cell growth rates were highly correlated for 89% of the years (**Supplementary Table S2**). Contrary to what we observed at the other sites, the correlation values at MIR varied considerably and were occasionally strongly negative (2015) (**Supplementary Table S2**). On average, the cambial cell growth rate was highest for the first cells of the tree ring, being 0.5 at all sites—except at SIM where it was 0.6—and decreased to 0.1 for the final cells (**Figure 6**). The difference between the predicted cambial cell growth rate and the observed cell growth rates ranged between 0.3 (BER) and 0.4 (DAN), having a systematic overestimation of the predicted values. The overestimation of the cell growth rates occurred for the first cells at all the sites in particular and became smaller when cell growth slowed (**Figure 6**). Inter-annual variations of the model predictions were similar to those of our observations (**Figure 6**). Accordingly, a greater variation of the intra-annual growth rate predicted in MIR matched with a greater variation in observed values (**Figure 6**). Regardless, the greater variability in growth rates in MIR could also be linked to the reduced number of available observations for this last site.

## Discussion

Understanding the effect of the environmental drivers on black spruce growth is crucial for predicting how environmental change will affect wood formation for this economically and ecologically important species. In Canada, correlations between tree-ring growth and the temperature and precipitation follow two different gradients, unraveling complex patterns in black spruce growth responses to these environmental factors (Nicault et al., 2015). Correlation with temperature is strongest in the North, whereas tree growth is most correlated with precipitation in the Western Canada (Huang et al., 2010; Walker and Johnstone, 2014). Correlations of tree growth with temperature and precipitation have also been confirmed by dendro-anatomical analyses at the cellular scale and have demonstrated that environmental factors affect xylem conductivity and long-term patterns of intra-annual cell traits (Puchi et al., 2019). According to the existing literature, the correlation between temperature and tree-ring growth is positive when temperature represents the most limiting factor and is negative in response to warming during the previous growing season and the current spring—probably reflecting drought stress (Huang et al., 2010; Walker and Johnstone, 2014; Nicault et al., 2015; Puchi et al., 2019). Correlation with precipitation, when significant, is always positive (Walker and Johnstone, 2014; Girardin et al., 2016; Puchi et al., 2019).

The variation in the local responses to environmental factors might indicate a long-term effect of the environmental factors, which is consistent with the conservative black spruce growth strategy (Chen et al., 2019). In this sense, VS model simulations improvements targeted on black spruce and VS model application on a larger territory could disentangle the common drivers of black spruce adaptation to environmental factors and their effect on tree-ring growth and wood formation at local scale. According to Vaganov et al. (2006), the algorithms underlying the VS model are based on the assumptions that intra-seasonal dynamics are the result of current environments, while the variability in cell production is determined by long-term conditions experienced by the trees, which are integrated to model’s simulations by parameterization. The effect of previous years conditions on growth is thus implicit, but the model has never been formulated to split the carryover effect of the environmental factors from the effect of the current environmental conditions.

The partial growth rates for the three main environmental factors predicted by the VS model revealed that different limiting factors shape tree-ring production in black spruce. At the beginning of the growing season, which is predicted by VS model for the end of May, temperature is the most limiting factor at all the sites. In cold environments, temperature is the major limiting factor of tree growth. Mean air temperature thresholds during xylogenesis range between 6 and 8°C (Rossi et al., 2007). Accordingly, the average temperature of the lower range of optimal temperatures for our sites (T_opt1_, T_min_), as estimated by the parameters, was between 6 and 7.6°C (**Supplementary Table S1**). The predicted end of the growing season occurred around the middle of September when photoperiod became the most limiting factor. In boreal and temperate forests, short days induce cambium dormancy by triggering the physiological mechanisms that lead to growth cessation (Buttò et al., 2020). At high latitudes, the shortening of day length also allows trees to anticipate lower temperatures and induces cold acclimation responses (Wingler, 2015).

The growth rate determined by water, i.e., water growth rate, was never a limiting factor along the gradient—except at BER where the predicted soil water content was heavily underestimated. This result confirms both field and greenhouse observations that the cell production and cell trait sizes of black spruce in eastern Canada do not change significantly with changes in soil moisture (Belien et al., 2012; Balducci et al., 2016). In contrast, Girardin et al. (2016) observed that stands growing in western Canadian ecoregions are very responsive to variations in soil water availability; this affects tree productivity by reducing the capacity of black spruce to fix carbon (Girardin et al., 2016). Thus, the lack of response of black spruce growth to variations in soil moisture must be ascribed to site conditions, which were well represented by the water growth rate of the VS model.

### Site Conditions

The best model performances in terms of prediction of tree-ring width, soil water content, as well as the onset and end of the growing season were obtained at the northernmost site (MIR), one of the driest sites along the gradient. These results confirm that the VS model parameter specification is based mainly on sites characterized by very cold conditions, such as in Siberia or on the Tibetan Plateau (Vaganov et al., 2011; He et al., 2017) and semi-arid or arid conditions, such as those in the southwestern US or northern Africa (Evans et al., 2006; Touchan et al., 2012). Therefore, the VS model ensures better simulations in dry environments, where it has been successfully applied to the study of the long-term response of tree growth to climate (Anchukaitis et al., 2006; Touchan et al., 2012; Zhang et al., 2016; Yang et al., 2017). However, our application of the VS model also provides useful guidance for improving model parameterization and its performance for wetter sites by involving the boreal forest characteristics of soil water balance and soil properties. According to the assumptions of the VS model, soil water content—deemed as the water available for tree growth—depends on precipitation, snowmelt, evaporation, and runoff (Vaganov et al., 2011). In the Canadian boreal forest, however, soil water balance and, in general, water and nutrient distribution within the soil horizons, are correlated strongly with organic layer composition and thickness—boreal soils can reach a depth of 150 cm (Simard et al., 2007; Laamrani et al., 2014). The forest floor of black spruce stands is composed generally of mosses and lichens, which modify the soil thermal conductivity and water content (O’Donnell et al., 2009). Due to its thickness and species-specific composition, the forest floor promotes water retention in the surface soil, thereby acting as a thermal buffer between the atmosphere and soil to decrease thermal conductivity (Sharratt, 1997; Turetsky et al., 2010). Accumulation on the forest floor entails a drop in soil temperature, and as such the decomposition rate of the organic matter slows; these conditions result in decreased tree growth (Fenton et al., 2005; Lavoie et al., 2005).

All soils at our study sites are podzols, although they differ in terms of depth to bedrock, soil thickness, and species composition on the forest floor. These peculiarities likely explain the mismatch between precipitation and soil water content that we observed along the gradient between 2002 and 2016. Despite receiving less precipitation, the southernmost sites of BER and SIM remained the wettest of the gradient. SIM is characterized by a thin organic layer (10–20 cm thick) and a very shallow bedrock (Rossi et al., 2016). As mentioned previously, the soil organic layer can negatively affect tree growth in boreal forest stands. However, the thickness of the organic layer at SIM was too thin to affect tree productivity (Lavoie et al., 2007); thus, the site at SIM is particularly favorable to black spruce growth. The other sites had deeper soils, 20–40 cm in depth, and differed in their forest floor characteristics (Rossi et al., 2016). The presence of mosses and a deeper soil at MIS and DAN foster a greater soil retention in the upper layer of the soil. In contrast, MIR has a forest floor dominated by lichens and contains a very thin soil depth; these properties favor the establishment of a thinner and drier forest floor (Waldron et al., 2013).

In this study, predictions of soil water content have improved *via* the activation of the “soil melting block” in the VS model, a specific module of the model that considers the contribution of snowmelt to soil moisture at the beginning of the growing season (Shishov et al., 2016). Rossi et al. (2011) had previously highlighted the importance of snowmelt at our sites by observing the effects of snowmelt on the duration of xylogenesis and cell production. The presence of accumulated snow prevents soil warming in the spring, and these colder temperatures inhibit root reactivation, delaying spring rehydration and, in turn, cambial reactivation (Turcotte et al., 2009). A later snowmelt corresponds to delays in soil warming, cambium activation, and cell production (Vaganov et al., 1999). Thus, the date of snowmelt influences the timing of wood formation and may explain some of the difference between the observed and the predicted onset of the growing season.

### Timing of Cell Division and Differentiation

Compared to the observed data, the predicted timings of cell division and enlargement were delayed from a few days to two weeks. This overestimation was more constant for the timings of cell division, and gradually higher across the tree ring for the timings of cell enlargement. For this, we advance the hypothesis that the upward shift of the predicted timings of cell division relative to observations can be improved by a more precise prediction of the start of the growing season. The increased mismatch between the predicted and observed timing of enlargement can be reduced further by including some information. Popkova et al. (2018) illustrate that the time that dividing cells spend in the cambial zone can be determined by splitting the predicted growing season into a number of periods equal to cell production. To leave the cambium, each cell must attain an average growth rate computed by the ratio between the cumulative sum of the daily growth rate obtained by VS model and cell production. However, to obtain a more reliable reconstruction of the temporal dynamics of cell division, a new assumption can be added to the model, one that considers the natural variation of cell production rates over the growing season. Furthermore, an accurate estimate of the number and order of cells entering the xylem should account for the number of cells that will never differentiate, i.e., the number of dormant cambial cells that constitute the mother cells and the cambial cells that undergo phloem differentiation (Plomion et al., 2001).

Cell production rates, depending by cambial activity, interact with the residence time of each cell within the differentiation zones. Variations in these cell production rates stem from the complex seasonal patterns that occur during tree-ring formation (Cuny et al., 2013; Balducci et al., 2016). An improved performance of the VS model timing procedure can be achieved by considering the specific pattern of the cell production seasonal rate. Cell production rate is characterized by a bell-shaped curve, with the peak and following decrease being in response to intra-seasonal dynamics and internal factors (Deslauriers and Morin, 2005). The peak in the cambial growth rate i.e. the number of cells produced per time units, matches a peak of auxin, the main hormone promoting cell division, and occurs during earlywood formation (Buttò et al., 2020). At the same time, soil water content and temperature affect cambial growth rate and cell production, the first prevailing in dry environments and the second in boreal climates (Deslauriers et al., 2008; Ziaco et al., 2018; Ren et al., 2019).

### VS Tree-Ring Growth Rate and Cell Differentiation

We compared cell growth rates with the cambial cell growth rates predicted by the protocol of Popkova et al. (2018). In particular, we tested the possibility of inferring the cell growth rate from the tree-ring growth curve obtained by the VS model by adjusting the procedure of Popkova et al. (2018) by focusing on the differentiation zones rather than the cambial zone. Once cell production is known, Popkova et al. (2018) infer the cambial cell growth rate using the curve of tree-ring growth rate and perform the most suitable regression between cambial cell growth rates and the measured cell diameters from actual tracheidograms. With the coefficients from the abovementioned regression, Popkova et al. (2018) then obtain those parameters useful for producing synthetic tracheidograms. However, cell diameter can be computed without using observations by integrating the relationship between cell traits and their temporal dynamics of differentiation (Cuny et al., 2014; Buttò et al., 2019). The timings of enlargement computed by the protocol proposed by Popkova et al. (2018), identify the start of cell differentiation i.e. the timings of cell enlargement, which is the day in which a cell starts increasing in size (**Table 2**). Xylem cell differentiation temporal dynamics assessment requires the computation of the timings of secondary wall deposition and cell maturation to estimate the duration of cell enlargement and the duration of secondary wall deposition. The duration of these differentiation phases depends on exogenous factors i.e. photoperiod, soil water content and temperature, which are already included in the model, and on endogenous factors i.e. hormonal signaling, sugars concentration (Rathgeber, 2017; Cartenì et al., 2018; Buttò et al., 2020). Then, a new framework that considers the nonlinear relationship between the duration of cell enlargement and cell diameter can be implemented, which also includes the effect of the secondary wall on the process of cell expansion (Buttò et al., 2019). During cell differentiation, secondary wall deposition influences cell diameter by altering the plastic properties of cell walls. Cell wall thickening, which occurs during cell trait differentiation, constrains cell expansion, especially during latewood formation when cell deposition lasts longer than during earlywood formation (Cartenì et al., 2018). These new findings that assess the quantitative relationships between cell traits, developmental temporal dynamics, and environmental factors should be implemented into the next versions of the VS model to improve simulations (Ziaco, 2020).

### The End of the Growing Season

The end of growth was one of the most critical portions for our comparisons, and in a wider sense, for studies of both xylem and bud phenology (Gallinat et al., 2015). At our sites, the end of wood formation occurred latest at the southernmost sites and showed a clear latitudinal pattern. Among the climatic factors, photoperiod and temperature are the main known drivers of the end of the wood formation, and the end of growth occurred earliest at the northernmost sites (Rossi et al., 2014; Cuny et al., 2019). However, other factors may also affect the end of the growing season, including cell production and sugar availability, the latter being crucial for cell wall deposition. Cell wall thickening at the end of the growing season lasts 30–40 days after the last cell is differentiated and drives the timing of latewood formation (Deslauriers et al., 2016; Cartenì et al., 2018). Cell production, which is the result of cambial activity, also influences the kinetics of wood formation, since competition for resources increases with the number of cells, and sugar availability shapes cell traits by modifying their differentiation phases (Cartenì et al., 2018). The end of the growing season can be parameterized during VS model calibration thought the critical growth rate, a coefficient representing the threshold under which the integral growth rate of the VS model does not allow wood formation anymore. Being based on the integration of the three partial growth rates linked to the environmental factors, the critical growth rate may not be sufficient for establishing a realistic end of the growing season, a moment that is not highly dependent on weather conditions, unlike at the start of the growing season (Rossi et al., 2012).

## Conclusions

In this study, we compared the predictions of intra-annual tree-ring formation dynamics estimated by the Vaganov–Shashkin (VS) model with field observations based on a 15 years-long monitoring of xylogenesis across a latitudinal gradient. Our results show that the model successfully describes the influence of climate variables on black spruce tree-ring growth. However, algorithms can still be improved to ensure a more reliable estimate of the timing of wood formation. Considering that the model has been tested for the first time on the Canadian boreal forest, it is difficult to relate the mismatch between predictions and observations to issues linked to the use of the model on wet environments or to the underlying algorithms computing the timings of cell development. VS-model parameterization is indeed based on semi-dry, dry or permafrost environments, which better support the assumptions of a constant soil water content during dormancy and of a closer relationship between tree growth and water availability at growth resumption. In our case, we tested the model on wet sites, and observed a low representativeness of the soil water content in spring, leading to cumulative errors in soil water estimation during the growing season. A recalibration of the model on other wet sites could provide more robust elements to improve the performance in terms of predicted water content and partial growth rate. Moreover, the algorithms computing the timings of cell production should consider that the cambial growth rate varies during the growing season with consequent effects on the residence time of each cell within the cambial zone. The development of a new procedure must involve phenology and dynamics of each cell during development, based on the underlying biological processes. As a consequence of cell differentiation dynamics, the computation of the cell traits, i.e. cell diameter and cell wall thickness, should be included in form of new module of the VS model based on larger datasets, encompassing species from different environments to obtain more extensive and exhaustive growth simulations.

## Data Availability Statement

The raw data supporting the conclusions of this article will be made available by the authors, without undue reservation.

## Author Contributions

VB, SR, and VS conceived and planned the study. VB carried out the analysis and wrote the manuscript. MP provided the initial version of the script to compute the timing of wood formation with the VS model. MP, IT, and VS provided technical support for the analyses. VB, VS, MP, IT, SR, AD, MH, and HM contributed to the interpretation of the results. All authors contributed to the article and approved the submitted version.

## Funding

This work was funded by the NSERC Industrial Research Chair on Black Spruce Growth and the Influence of Spruce Budworm on Landscape Variability in Boreal Forests, the Canada Foundation for Innovation, le Consortium de Recherche sur la Forêt Boréale Commerciale, les Fonds de Recherche sur la Nature et les Technologies du Québec, and la Forêt d’Enseignement et de Recherche Simoncouche. VS, MP, and IT were supported by the Russian Ministry of Science and Higher Education (projects #FSRZ-2020-0010 and #FSRZ-2020-0014).

## Conflict of Interest

The authors declare that the research was conducted in the absence of any commercial or financial relationships that could be construed as a potential conflict of interest.

The reviewer [BY] declared a shared affiliation, though no other collaboration, with one of the authors [SR] to the handling Editor.

## References

[B1] Anchukaitis K. J. Evans M. N. Kaplan A. Vaganov E. A. Hughes M. K. Grissino-Mayer H. D. (2006). Forward modeling of regional scale tree-ring patterns in the southeastern United States and the recent influence of summer drought. Geophys. Res. Lett. 33, 2–5. 10.1029/2005GL025050

[B2] Anchukaitis K. J. Evans M. N. Hughes M. K. Vaganov E. A. (2020). An interpreted language implementation of the Vaganov–Shashkin tree-ring proxy system model. Dendrochronologia 60, 125677. 10.1016/j.dendro.2020.125677

[B3] Balducci L. Cuny H. E. Rathgeber C. B. K. Deslauriers A. Giovannelli A. Rossi S. (2016). Compensatory mechanisms mitigate the effect of warming and drought on wood formation. Plant Cell Environ. 39, 1338–1352. 10.1111/pce.12689 26662380

[B4] Belien E. Rossi S. Morin H. Deslauriers A. (2012). Xylogenesis in black spruce subjected to rain exclusion in the field. Can. J. For. Res. 42, 1306–1315. 10.1139/x2012-095

[B5] Bunn A. G. (2008). A dendrochronology program library in R (dplR). Dendrochronologia 26, 115–124. 10.1016/j.dendro.2008.01.002

[B6] Buttò V. Deslauriers A. Rossi S. Rozenberg P. Shishov V. Morin H. (2020). The role of plant hormones in tree-ring formation. Trees 1–21. 10.1007/s00468-019-01940-4

[B7] Buttò V. Rossi S. Deslauriers A. Morin H. (2019). Is size an issue of time? Relationship between the duration of xylem development and cell traits. Ann. Bot. 123, 1257–1265. 10.1093/aob/mcz032 30873532 PMC6612947

[B8] Cartenì F. Deslauriers A. Rossi S. Morin H. De Micco V. Mazzoleni S. (2018). The physiological mechanisms behind the earlywood-to-latewood transition: a process-based modelling approach. Front. Plant Sci. 9, 1053. 10.3389/fpls.2018.01053 30079078 PMC6063077

[B9] Chen L. Rossi S. Deslauriers A. Liu J. (2019). Contrasting strategies of xylem formation between black spruce and balsam fir in Quebec, Canada. Tree Physiol. 39, 747–754. 10.1093/treephys/tpy151 30715531

[B10] Cook E. R. Pederson N. (2011). “Uncertainty, Emergence, and Statistics in Dendrochronology,” in Dendroclimatology: progress and prospects. (Dordrecht: Springer) 77–112. 10.1007/978-1-4020-5725-0_4

[B11] Cook E. R. Shiyatov S. G. Mazepa V. S. Ecology A. Branch U. (1990). Methods of Dendrochronology: Tree-ring standardization and growth-trend estimation. Methods Dendrochronol. Appl. Environ. Sci., 104–123. 10.1007/978-94-015-7879-0

[B12] Cuny H. E. Rathgeber C. B. K. K. Kiessé T. S. Hartmann F. P. Barbeito I. Fournier M. (2013). Generalized additive models reveal the intrinsic complexity of wood formation dynamics. J. Exp. Bot. 64, 1983–1994. 10.1093/jxb/ert057 23530132 PMC3638824

[B13] Cuny H. E. Rathgeber C. B. K. B. K. Frank D. Fonti P. Fournier M. (2014). Kinetics of tracheid development explain conifer tree-ring structure. New Phytol. 203, 1231–1241. 10.1111/nph.12871 24890661

[B14] Cuny H. E. Fonti P. Rathgeber C. B. K. Arx G. Peters R. L. Frank D. C. (2019). Couplings in cell differentiation kinetics mitigate air temperature influence on conifer wood anatomy. Plant Cell Environ. 42, 1222–1232. 10.1111/pce.13464 30326549

[B15] Danis P.-A. Hatté C. Misson L. Guiot J. (2012). MAIDENiso: a multiproxy biophysical model of tree-ring width and oxygen and carbon isotopes. Can. J. For. Res. 42, 1697–1713. 10.1139/X2012-089

[B16] Deslauriers A. Morin H. (2005). Intra-annual tracheid production in balsam fir stems and the effect of meteorological variables. Trees 19, 402–408. 10.1007/s00468-004-0398-8

[B17] Deslauriers A. Morin H. Begin Y. (2003). Cellular phenology of annual ring formation of *Abies balsamea* in the Quebec boreal forest (Canada). Can. J. For. Res. 33, 190–200. 10.1139/x02-178

[B18] Deslauriers A. Rossi S. Anfodillo T. Saracino A. (2008). Cambial phenology, wood formation and temperature thresholds in two contrasting years at high altitude in southern Italy. Tree Physiol. 28, 863–871. 18381267 10.1093/treephys/28.6.863

[B19] Deslauriers A. Huang J.-G. Balducci L. Beaulieu M. Rossi S. (2016). The contribution of carbon and water in modulating wood formation in black spruce saplings. Plant Physiol. 170, 01525. 10.1104/pp.15.01525 PMC4825115 26850274

[B20] Evans M. N. Reichert B. K. Kaplan A. Anchukaitis K. J. Vaganov E. A. Hughes M. K. (2006). A forward modeling approach to paleoclimatic interpretation of tree-ring data. J. Geophys. Res. Biogeosci. 111, G03008. 10.1029/2006JG000166

[B21] Evans M. E. K. Gugger P. F. Lynch A. M. Guiterman C. H. Fowler J. C. Klesse S. (2018). Dendroecology meets genomics in the common garden: new insights into climate adaptation. New Phytol. 218, 401–403. 10.1111/NPH.15094 29561071

[B22] Fenton N. Lecomte N. Légaré S. Bergeron Y. (2005). Paludification in black spruce (Picea mariana) forests of eastern Canada: Potential factors and management implications. For. Ecol. Manage. 213, 151–159. 10.1016/j.foreco.2005.03.017

[B23] Fonti P. Von Arx G. García-González I. Eilmann B. Sass-Klaassen U. Gärtner H. (2010). Studying global change through investigation of the plastic responses of xylem anatomy in tree rings. New Phytol. 185, 42–53. 10.1111/j.1469-8137.2009.03030.x 19780986

[B24] Gallinat A. S. Primack R. B. Wagner D. L. (2015). Autumn, the neglected season in climate change research. Trends Ecol. Evol. 30, 169–176. 10.1016/j.tree.2015.01.004 25662784

[B25] Girardin M. P. Hogg E. H. Bernier P. Y. Kurz W. A. Guo X. J. Cyr G. (2016). Negative impacts of high temperatures on growth of black spruce forests intensify with the anticipated climate warming. Glob. Change Biol. 22, 627–643. 10.1111/gcb.13072 26507106

[B26] Hacke U. G. Spicer R. Schreiber S. G. Plavcová L. (2017). An ecophysiological and developmental perspective on variation in vessel diameter. Plant Cell Environ. 40, 831–845. 10.1111/pce.12777 27304704

[B27] He M. Shishov V. Kaparova N. Yang B. Bräuning A. Grießinger J. (2017). Process-based modeling of tree-ring formation and its relationships with climate on the Tibetan Plateau. Dendrochronologia 42, 31–41. 10.1016/j.dendro.2017.01.002

[B28] Hopkinson R. F. Mckenney D. W. Milewska E. J. Hutchinson M. F. Papadopol P. Vincent A. L. A. (2011). Impact of aligning climatological day on gridding daily maximum-minimum temperature and precipitation over Canada. J. Appl. Meteorol. Climatol. 50, 1654–1665. 10.1175/2011JAMC2684.1

[B29] Huang J. A. Tardif J. C. Bergeron Y. Denneler B. Berninger F. Girardin M. P. (2010). Radial growth response of four dominant boreal tree species to climate along a latitudinal gradient in the eastern Canadian boreal forest. Glob. Change Biol. 16, 711–731. 10.1111/j.1365-2486.2009.01990.x

[B30] Hutchinson M. F. Mckenney D. W. Lawrence K. Pedlar J. H. Hopkinson R. F. Milewska E. (2009). Development and Testing of Canada-Wide Interpolated Spatial Models of Daily Minimum-Maximum Temperature and Precipitation for 1961-2003. J. Appl. Meteorol. Climatol. 48 (4), 725–741. 10.1175/2008JAMC1979.1

[B31] James G. Witten D. Hastie T. Tibshirani R. (2013). An introduction to statistical learning. Available at: https://link.springer.com/content/pdf/10.1007/978-1-4614-7138-7.pdf (Accessed June 11, 2020).

[B32] Kaplan D. Pruim R. (2020). ggformula: Formula Interface to the Grammar of Graphics. Available at: https://github.com/ProjectMOSAIC/ggformula/issues (Accessed June 11, 2020).

[B33] Laamrani A. Valeria O. Bergeron Y. Fenton N. Cheng L. Z. Anyomi K. (2014). Effects of topography and thickness of organic layer on productivity of black spruce boreal forests of the canadian clay belt region. For. Ecol. Manage. 330, 144–157. 10.1016/j.foreco.2014.07.013

[B34] Lavoie M. Paré D. Fenton N. Groot A. Taylor K. (2005). Paludification and management of forested peatlands in Canada: A literature review. Environ. Rev. 13, 21–50. 10.1139/a05-006

[B35] Lavoie M. Harper K. Paré D. Bergeron Y. (2007). Spatial pattern in the organic layer and tree growth: A case study from regenerating *Picea mariana* stands prone to paludification. J. Veg. Sci. 18, 213–222. 10.1111/j.1654-1103.2007.tb02532.x

[B36] McKenney D. W. Hutchinson M. F. Papadopol P. Lawrence K. Pedlar J. Campbell K. (2011). Customized spatial climate models for Canada. Bull. Am. Meteorol. Soc 92, 1611–1622. 10.1175/2011BAMS3132.1

[B37] Nicault A. Boucher E. Tapsoba D. Arseneault D. Berninger F. Bégin C. (2015). Spatial analysis of black spruce (Picea mariana (Mill.) B.S.P.) radial growth response to climate in northern Québec – Labrador Peninsula, Canada. Can. J. For. Res. 45, 343–352. 10.1139/cjfr-2014-0080

[B38] O’Donnell J. A. Romanovsky V. E. Harden J. W. McGuire A. D. (2009). The effect of moisture content on the thermal conductivity of moss and organic soil horizons from black spruce ecosystems in interior alaska. Soil Sci. 174, 646–651. 10.1097/SS.0b013e3181c4a7f8

[B39] Plomion C. Leprovost G. Stokes A. (2001). Wood Formation in Trees Wood Formation in Trees. Plant Physiol. 127, 1513–1523. 10.1104/pp.010816.1 11743096 PMC1540185

[B40] Popkova M. II Vaganov E. A. Shishov V. V. Babushkina E. A. Rossi S. Fonti M. V. (2018). Modeled tracheidograms disclose drought influence on Pinus sylvestris tree-rings structure from Siberian forest-steppe. Front. Plant Sci. 9, 1144. 10.3389/fpls.2018.01144 30127799 PMC6088211

[B41] Puchi P. F. Castagneri D. Rossi S. Carrer M. (2019). Wood anatomical traits in black spruce reveal latent water constraints on the boreal forest. Glob. Change Biol. 26 (3), 1767–1777. 10.1111/gcb.14906 31692158

[B42] Rathgeber C. B. K. (2017). Conifer tree-ring density inter-annual variability - anatomical, physiological and environmental determinants. New Phytol. 216, 621–625. 10.1111/nph.14763 29034974

[B43] Ren P. Ziaco E. Rossi S. Biondi F. Prislan P. Liang E. (2019). Growth rate rather than growing season length determines wood biomass in dry environments. Agric. For. Meteorol. 271, 46–53. 10.1016/j.agrformet.2019.02.031

[B44] Rossi S. Menardi R. Anfodillo T. (2006). Trephor: a new tool for sampling microcores from tree stems. IAWA J. 27, 89–97. 10.1163/22941932-90000139

[B45] Rossi S. Deslauriers A. Anfodillo T. Carraro V. (2007). Evidence of threshold temperatures for xylogenesis in conifers at high altitudes. Oecologia 152, 1–12. 10.1007/s00442-006-0625-7 17165095

[B46] Rossi S. Morin H. Deslauriers A. (2011). Multi-scale influence of snowmelt on xylogenesis of black spruce. Arct. Antarct. Alp. Res. 43 (3), 457–464.

[B47] Rossi S. Morin H. Deslauriers A. (2012). Causes and correlations in cambium phenology: Towards an integrated framework of xylogenesis. J. Exp. Bot. 63, 2117–2126. 10.1093/jxb/err423 22174441 PMC3295399

[B48] Rossi S. Girard M.-J. J. Morin H. (2014). Lengthening of the duration of xylogenesis engenders disproportionate increases in xylem production. Glob. Change Biol. 20, 2261–2271. 10.1111/gcb.12470 24259354

[B49] Rossi S. Couture É. Plante X. Morin H. (2016). Fine roots and ectomycorrhizal colonization in black spruce subjected to reductions in soil moisture. Botany 94, 23–30. 10.1139/cjb-2015-0093

[B50] Rossi S. (2015). Local adaptations and climate change: converging sensitivity of bud break in black spruce provenances. Int. J. Biometeorol. 59, 827–835. 10.1007/s00484-014-0900-y 25225116

[B51] Sharratt B. S. (1997). Thermal conductivity and water retention of a black spruce forest floor. Soil Sci. 162, 576–582. 10.1097/00010694-199708000-00006

[B52] Shishov V. V. Tychkov I. II Popkova M. II Ilyin V. A. Bryukhanova M. V. Kirdyanov A. V. (2016). VS-oscilloscope: A new tool to parameterize tree radial growth based on climate conditions. Dendrochronologia 39, 42–50. 10.1016/j.dendro.2015.10.001

[B53] Simard M. Lecomte N. Bergeron Y. Bernier P. Y. Paré D. (2007). Forest productivity decline caused by successional paludification of boreal soils. Ecol. Appl. 17, 1619–1637. 10.1890/06-1795.1 17913128

[B54] Skene D. S. (1969). The Period of Time Taken by Cambial Derivatives to Grow and Differentiate into Tracheids in Pinus radiata: D. Don. Ann. Bot. 33, 253–262. 10.1093/oxfordjournals.aob.a084280

[B55] Touchan R. Shishov V. V. Meko D. M. Nouiri I. Grachev A. (2012). Process based model sheds light on climate sensitivity of Mediterranean tree-ring width. Biogeosciences 9, 965–972. 10.5194/bg-9-965-2012

[B56] Turcotte A. Morin H. Krause C. Deslauriers A. Thibeault-Martel M. (2009). The timing of spring rehydration and its relation with the onset of wood formation in black spruce. Agric. For. Meteorol. 149, 1403–1409. 10.1016/j.agrformet.2009.03.010

[B57] Turetsky M. R. Mack M. C. Hollingsworth T. N. Harden J. W. (2010). The role of mosses in ecosystem succession and function in Alaska’s boreal forest. Can. J. For. Res. 40, 1237–1264. 10.1139/X10-072

[B58] Tychkov I. II Sviderskaya I. V. Babushkina E. A. Popkova M. II Vaganov E. A. Shishov V. V. (2019). How can the parameterization of a process-based model help us understand real tree-ring growth? Trees - Struct. Funct. 33, 345–357. 10.1007/s00468-018-1780-2

[B59] Vaganov E. A. Hughes M. K. Kirdyanov A. V. Schweingruber F. H. Silkin P. P. (1999). Influence of snowfall and melt timing on tree growth in subarctic Eurasia. Nature 400, 149–151. 10.1038/22087

[B60] Vaganov E. A. Evgeniĭ A. Hughes M. K. Shashkin A. V. Aleksandr V. Hughes M. K. (2006). Growth Dynamics of Conifer Tree Rings: Images of Past and Future Environments. Ecological (Springer-Verlag Berlin: Springer). 10.1086/586955

[B61] Vaganov E. A. Anchukaitis K. J. Evans M. N. (2011). “How Well Understood Are the Processes that Create Dendroclimatic Records? A Mechanistic Model of the Climatic Control on Conifer Tree-Ring Growth Dynamics,” in Dendroclimatology. Developments in Paleoenvironmental Research (Dordrecht: Springer), pp 37–75. 10.1007/978-1-4020-5725-0_3

[B62] Waldron K. Ruel J. C. Gauthier S. (2013). The effects of site characteristics on the landscape-level windthrow regime in the North Shore region of Quebec, Canada. Forestry 86, 159–171. 10.1093/forestry/cps061

[B63] Walker X. Johnstone J. F. (2014). Widespread negative correlations between black spruce growth and temperature across topographic moisture gradients in the boreal forest. Environ. Res. Lett. 9:64016. 10.1088/1748-9326/9/6/064016

[B64] Wingler A. (2015). Comparison of signaling interactions determining annual and perennial plant growth in response to low temperature. Front. Plant Sci. 5:794. 10.3389/fpls.2014.00794 25628637 PMC4290479

[B65] Yang B. He M. Shishov V. Tychkov I. Vaganov E. Rossi S. (2017). New perspective on spring vegetation phenology and global climate change based on Tibetan Plateau tree-ring data. Proc. Natl. Acad. Sci. U. S. A. 114, 6966–6971. 10.1073/pnas.1616608114 28630302 PMC5502585

[B66] Zhang J. Gou X. Zhang Y. Lu M. Xu X. Zhang F. (2016). Forward modeling analyses of Qilian Juniper (Sabina przewalskii) growth in response to climate factors in different regions of the Qilian Mountains, northwestern China. Trees - Struct. Funct. 30, 175–188. 10.1007/s00468-015-1286-0

[B67] Ziaco E. Truettner C. Biondi F. Bullock S. (2018). Moisture-driven xylogenesis in Pinus ponderosa from a Mojave Desert mountain reveals high phenological plasticity. Plant Cell Environ. 41, 823–836. 10.1111/pce.13152 29361193

[B68] Ziaco E. (2020). A phenology-based approach to the analysis of conifers intra-annual xylem anatomy in water-limited environments. Dendrochronologia 59:125662. 10.1016/j.dendro.2019.125662

