# Supplementary material for: Comparing the Cell Dynamics of Tree-Ring Formation Observed in Microcores and as Predicted by the Vaganov–Shashkin Model
Source: Front Plant Sci. 2020 Aug 14;11:1268. doi: 10.3389/fpls.2020.01268 (PMC7457011; doi:10.3389/fpls.2020.01268)
Supplement: Supplementary file 1 [file Table_1.docx]

**Supplementary materials**

**Figure S1** Standardized tree-ring width chronologies for the five study sites along the latitudinal gradient. Sites are organized according to latitude (the southernmost site (SIM) as the top row of graphs, the northernmost (MIR) is on the bottom row). *R* and RMSE are the Pearson correlation and the root mean squared error for each pair of curves, respectively.


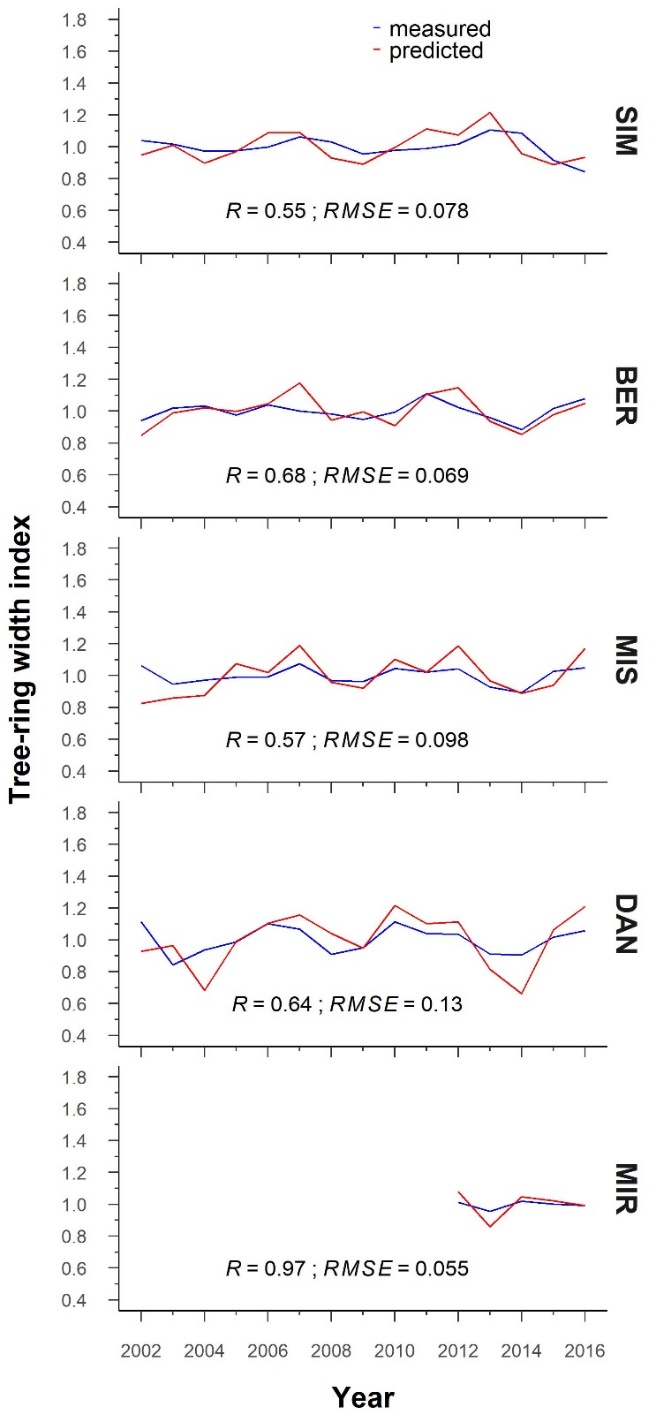
Table S1 Estimated VS model parameters for the five chronologies of the five sites along the latitudinal gradient.

| **Parameter** | **Description (units)** | **SIM** | **BER** | **MIS** | **DAN** | **MIR** |
| --- | --- | --- | --- | --- | --- | --- |
| **T_min_** | Minimum temperature for tree growth (°C) | 4 | 4 | 5 | 4 | 4 |
| **T_opt1_** | Lower end of the optimal temperature range (°C) | 8 | 7 | 8 | 9 | 11 |
| **T_opt2_** | Upper end of the optimal temperature range (°C) | 17 | 15 | 13 | 15 | 17 |
| **T_max_** | Maximum temperature for tree growth | 27 | 24 | 25 | 29 | 26 |
| **W_min_** | Minimum soil moisture for tree growth (V/Vs) | 0.0025 | 0.135 | 0.0125 | 0.0225 | 0.005 |
| **W_opt1_** | Lower end of the range of the optimal soil moisture (V/Vs) | 0.175 | 0.25 | 0.125 | 0.15 | 0.15 |
| **W_opt2_** | Upper end of the optimal soil moisture range (V/Vs) | 0.459 | 0.3 | 0.375 | 0.45 | 0.325 |
| **W_max_** | Growth is stopped at this soil moisture (V/Vs) | 0.525 | 0.675 | 0.6 | 0.6 | 0.45 |
| **W_0_** | Initial soil moisture (V/Vs) | 0.4 | 0.3 | 0.14 | 0.2 | 0.25 |
| **T_beg_** | Sum of temperature at the start growth (°C) | 115 | 105 | 125 | 120 | 130 |
| **lr** | Depth of the root system (mm) | 150 | 200 | 150 | 200 | 200 |
| **P_max_** | Maximum daily precipitation for a saturated soil (mm/day) | 24 | 46 | 22 | 24 | 35 |
| **C_1_** | Fraction of precipitation penetrating the soil (not captured by the canopy) | 0.06 | 0.17 | 0.14 | 0.15 | 0.17 |
| **C_2_** | First coefficient for calculating transpiration (mm/day) | 0.0725 | 0.14 | 0.0925 | 0.12 | 0.125 |
| **C_3_** | Second coefficient for calculating transpiration (mm/day) | 0.09 | 0.12 | 0.13 | 0.115 | 0.145 |
| **ᴧ** | Coefficient for water drainage from the soil (rel. unit) | 0.001 | 0.006 | 0.008 | 0.005 | 0.008 |
| **T_sm_** | Snowmelt sum of temperatures | 30 | 30 | 30 | 30 | 30 |
| **Sm_1_** | First coefficient for snowmelt | 12 | 9 | 8 | 10 | 13 |
| **Sm_2_** | Second coefficient for snowmelt | 0.0125 | 0.155 | 0.0065 | 0.012 | 0.0045 |
| **V_cr_** | Critical growth rate | 0.15 | 0.12 | 0.03 | 0.16 | 0.07 |

**Table S2** Intra-annual correlation between the predicted and observed variables for soil water content during dormancy and wood formation and the timing of both cell division and enlargement. Growth rate correlation was performed between the predicted cambial cell growth rate and the observed cell growth rate

| Site | r correlation | 2002 | 2003 | 2004 | 2005 | 2006 | 2007 | 2008 | 2009 | 2010 | 2011 | 2012 | 2013 | 2014 | 2015 | 2016 |
| --- | --- | --- | --- | --- | --- | --- | --- | --- | --- | --- | --- | --- | --- | --- | --- | --- |
| SIM | **Soil water content during dormancy** | 0.27 | -0.37 | 0 | 0.61 | -0.13 | -0.49 | -0.9 | 0.33 | 0.02 | -0.24 | -0.08 | 0.42 | -0.35 | -0.43 | 0.46 |
| BER |  | -0.2 | 0.43 | 0.12 | -0.2 | 0.47 | -0.33 | -0.05 | 0.68 | -0.3 | 0.31 | 0.3 | 0.11 | -0.23 | -0.02 | 0.45 |
| MIS |  | 0.01 | 0.28 | -0.87 | -0.82 | 0.64 | -0.66 | 0.86 | -0.52 | 0.51 | 0.26 | 0.62 | -0.53 | -0.59 | -0.76 | 0.55 |
| DAN |  | 0.48 | 0.52 | 0.68 | -0.24 | -0.16 | -0.24 | -0.88 | -0.04 | -0.14 | -0.12 | -0.19 | -0.11 | 0.38 | 0.3 | 0.16 |
| MIR |  |  |  |  |  |  |  |  |  |  |  | -0.46 | 0.06 | -0.6 | 0.7 | -0.59 |
| SIM | **Soil water content during wood formation** | 0.73 | 0.63 | 0.14 | 0.66 | 0.58 | -0.11 | -0.08 | 0.16 | 0.58 | -0.24 | 0.56 | 0.38 | 0.69 | 0.51 | 0.71 |
| BER |  | 0.55 | -0.04 | 0.73 | 0.89 | 0.82 | 0.26 | 0.88 | 0.42 | 0.93 | 0.92 | 0.86 | 0.55 | 0.93 | 0.58 | 0.86 |
| MIS |  | -0.04 | -0.64 | 0.51 | -0.49 | 0.43 | 0.41 | 0.01 | -0.73 | 0.17 | -0.39 | -0.08 | -0.45 | 0.35 | -0.58 | 0.45 |
| DAN |  | -0.08 | -0.21 | -0.26 | 0.14 | -0.25 | 0.35 | 0.02 | -0.01 | -0.24 | -0.21 | -0.1 | -0.57 | 0.2 | 0.07 | 0.38 |
| MIR |  |  |  |  |  |  |  |  |  |  |  | 0.64 | -0.11 | 0.31 | 0.64 | 0.32 |
| SIM | **Timing of cell division** | 0.96 | 0.99 | 0.99 | 0.99 | 0.99 | 1 | 0.98 | 0.96 | 1 | 0.97 | 0.99 | 1 | 0.99 | 0.97 | 0.95 |
| BER |  | 0.97 | 0.99 | 0.98 | 0.96 | 0.99 | 0.99 | 0.93 | 0.99 | 0.98 | 0.96 | 0.94 | 0.99 | 0.99 | 0.98 | 0.93 |
| MIS |  | 0.96 | 0.99 | 0.99 | 0.98 | 0.99 | 0.99 | 0.99 | 0.99 | 0.99 | 1 | 0.99 | 1 | 0.99 | 1 | 0.99 |
| DAN |  | 0.97 | 0.98 | 0.99 | 1 | 0.99 | 1 | 1 | 0.98 | 0.99 | 1 | 1 | 0.97 | 1 | 0.99 | 0.99 |
| MIR |  |  |  |  |  |  |  |  |  |  |  | 1 | 0.99 | 0.94 | 0.99 | 0.99 |
| SIM | **Timing of cell enlargement** | 0.98 | 0.99 | 0.99 | 1 | 1 | 1 | 0.99 | 0.98 | 1 | 0.98 | 0.99 | 1 | 0.99 | 0.99 | 0.94 |
| BER |  | 0.99 | 1 | 0.99 | 0.98 | 0.99 | 0.99 | 0.97 | 0.99 | 0.97 | 1 | 0.97 | 1 | 0.98 | 0.98 | 0.96 |
| MIS |  | 0.99 | 1 | 0.99 | 0.98 | 0.99 | 0.99 | 0.92 | 0.99 | 0.99 | 0.98 | 0.99 | 0.98 | 0.99 | 0.98 | 0.99 |
| DAN |  | 0.99 | 1 | 0.99 | 1 | 0.99 | 0.98 | 0.99 | 1 | 0.99 | 0.99 | 0.99 | 0.95 | 0.99 | 0.99 | 0.99 |
| MIR |  |  |  |  |  |  |  |  |  |  |  | 1 | 0.97 | 0.97 | 1 | 0.99 |
| SIM | **Growth rate** | 0.65 | 0.41 | 0.88 | 0.72 | 0.35 | 0.55 | 0.88 | 0.75 | 0.67 | 0.73 | 0.66 | 0.57 | 0.77 | 0.45 | 0.39 |
| BER |  | 0.78 | 0.87 | 0.54 | 0.67 | 0.71 | 0.83 | 0.96 | 0.66 | 0.93 | 0.72 | 0.53 | 0.23 | 0.33 | 0.47 | 0.91 |
| MIS |  | 0.74 | 0.64 | 0.42 | 0.49 | 0.16 | 0.56 | 0.63 | 0.74 | 0.73 | 0.21 | 0.6 | 0.29 | 0.56 | 0.7 | 0.77 |
| DAN |  | 0.79 | 0.79 | 0.65 | 0.44 | 0.75 | 0.91 | 0.88 | 0.87 | 0.68 | 0.82 | 0.88 | 0.85 | 0.39 | 0.95 | 0.68 |
| MIR |  |  |  |  |  |  |  |  |  |  |  | 0.72 | 0.6 | 0.5 | -0.63 | 0.36 |
